# Analysis of the wool thickness and medullation characteristics based on sex and color in a herd of alpacas in Poland

**DOI:** 10.5194/aab-64-157-2021

**Published:** 2021-05-05

**Authors:** Aurelia Radzik-Rant, Karolina Wiercińska

**Affiliations:** Warsaw University of Life Science – SGGW, Institute of Animal Sciences, Department of Animal Breeding, Ciszewskiego 8, 02 – 786 Warsaw, Poland

## Abstract

The objective of this study was to analyze the thickness and medullation characteristics of the wool of a herd of Huacaya alpacas kept in Poland. Wool samples were collected from 36 adult alpacas, including 22 females and 14 males. Light (15 animals) and dark (21 animals) color varieties of wool were considered in this research. A projection microscope was used to measure the fiber diameter and assess the medullation. Each fiber was categorized according to the medulla as a non-medullated, discontinuous medullated or continuous medullated fiber. The mean fiber diameter (MFD), standard deviation (SD), coefficient of variation (CV), comfort factor (CF) and prickling factor (PF) were determined for each sample. The MFD, SD and CV were also determined for the abovementioned fiber categories. The mean fiber diameter of all alpacas tested was 25.31 µm. The CF and PF were 77.79 % and 22.21 %, respectively. The medullation percentage in the wool of the study alpacas was 68.91 %. The mean fiber diameter was larger in males (P<0.05) than in females. There were no differences between males and females in terms of the proportion of fibers with a diameter < 30 µm (CF) and > 30 µm (PF). The non-medullated fibers in the wool of females were thinner (P<0.05) than in the wool of males. The wool of males also had a larger degree of medullation. Light wool was thinner (P<0.05) than dark wool. The discontinuous and the continuous medullated fibers were thicker (P<0.05) in dark wool than in light wool. In dark wool, the share of discontinuous fibers was higher (P<0.05) and the share of the non-medullated fibers was lower (P<0.05) than in light wool. The presence of various types of medulla or the absence of medulla was noted in fibers with smaller and larger diameters, regardless of the sex of the animals or the color of the wool.

## 1 Introduction

Due to the extraordinary quality of their wool fibers, alpacas are gaining popularity not only in South America but also on other continents, including Europe. The interest in breeding these animals in Poland has increased, although their population is small and only comprises about 2000 animals (Krajewska et al., 2020). Peruvian farmers keep alpacas for wool (fiber), meat and as a means of transportation, although their use as a source of wool has long been in a priority (Aylan-Parker and McGregor, 2002; Frank et al., 2006; Gutiérrez et al., 2009). Similarly, wool production is the main purpose of keeping these animals in Poland, although “alpacotherapy” and recreation are also of great interest.

**Figure 1:**
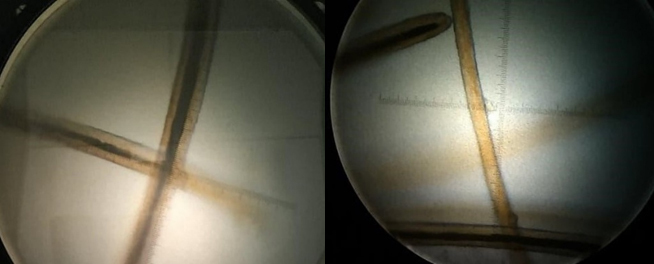
Fiber sample under a projection microscope during the fiber categorization process.

Worldwide, the quality of alpaca wool is considered to be among the best in the textile industry. However, despite its many advantages, alpaca wool (fiber) still differs with respect to delicacy from other thin animal fibers, such as cashmere (Süpüren et al., 2015). The main reason for this is the “prickling factor” associated with alpaca wool. This factor has been linked to fiber diameter, especially to fibers thicker than 30 µm. Therefore, the percentage of fibers with a diameter < 30 µm has been adopted as the “comfort factor” (McGregor, 1997; Frank et al., 2006). The discomfort (prickling factor) associated with alpaca wool is also attributed, as in sheep wool, to the medullated fibers commonly found in the fleece and to the type of medulla (Sánchez et al., 2016; Frank et al., 2014, 2017). The greatest objections are raised to fibers with a thickness greater than 25 µm that contain medullas which occupy more than 94 % of the fiber diameter (IWTO-57, 1998). Hence, regarding the improvement of alpaca wool quality, the aim is to reduce the thickness of the fibers, eliminate the medullated fibers and carry out selective breeding in order to obtain more non-medullated fibers in the fleece (Gutiérrez et al., 2011, 2014; Cruz et al., 2019). According to Pinares et al. (2018), there is a strong genetic link between medullation and fiber thickness; however, it is better to study this relationship individually in each fiber than to use global parameters.

The value of the fiber diameter and the degree of medullation depends on many factors, both genetic and phenotypic. The phenotypic factors influencing the abovementioned features include the sex of the animal and the color variety of the wool. However research in this area presents conflicting results: some researchers indicate that the fiber diameter is independent of sex, some report that the wool of males is thicker than that of females and some state that the wool of males is thinner than that of females (Wuliji, 2000; Lupton et al., 2006; McColl et al., 2004; Montes et al., 2008). The relationship between the color of the coat and the fiber diameter or coat color and medullation is also not always clear. Wuliji (2000) and Wurzinger et al. (2006) indicated that there is no relationship between wool thickness and color. In contrast, McGregor and Butler (2004), Lupton et al. (2006) and Cruz et al. (2017) reported that dark wool is thicker than light wool, whereas Frank et al. (2006) indicated that the opposite is true. Hoffman (2006) reported that the medullas in fibers from light alpacas cover up to 60 % of the fiber diameter, and McColl et al. (2004) stated this value is even higher. Both papers agree that the medullas are thinner in dark fleeces.

The aim of this study was a detailed analysis of the thickness and medullation characteristics of a herd of Huacaya alpacas kept in Poland with consideration of the sex and coat color of the animals.

## 2 Material and methods

The research material was the wool of Huacaya alpacas from a farm located in the province of Podlasie, Poland. The animals were kept under uniform environmental conditions with constant zootechnical and veterinary supervision. According to Polish law and EU Directive no. 2010/63/EU (European Parliament and the Council of the European Union, 2010), the experiment did not require approval from the local ethical committee because it was carried out on a private farm under production conditions.

The wool samples were collected from 36 adult alpacas, including 22 females and 14 males. The animals tested were characterized by two wool color categories: light (white and light beige) and dark (black and brown). In the group of females, 9 had a light-colored wool and 13 had dark-colored wool; in the group of males, 6 had light-colored wool and 8 had dark-colored wool. In total (from the 36 animals tested), 15 had light-colored wool and 21 had dark-colored wool.

Wool samples were collected just prior to annual shearing from the middle of the left side of the animal, behind the third rib, halfway between the back line and the belly line – the most representative area for evaluating the average diameter of alpaca wool fibers (Aylan-Parker and McGregor, 2002). About 10 g of wool was collected for each sample. The samples were sealed in plastic bags and stored until analysis in the laboratory.

Evaluation of the fiber diameter and medullation was carried out using a projection microscope in accordance with testing method IWTO-8 (2011). A minimum of 600 fibers were analyzed in each of the 36 samples, and a total of 21 600 fibers were analyzed. The choice of the measurement method was dictated by the possibility of determining the type of medulla (Fig. 1). Each fiber was classified according to the category of the medulla into non-medullated, discontinuous medullated and continuous medullated fibers.

**Table 1:** The overall characteristics of wool thickness and the medullation features of the alpacas investigated.

Item	Total fibers	Non-medullatedfibers	Discontinuousmedullated fibers	Continuousmedullated fibers
	LSM ± SE	LSM ± SE	LSM ± SE	LSM ± SE
Mean fiber diameter (MFD, µm)	25.31 ± 0.75	21.69 ± 0.70	25.89 ± 0.71	30.53 ± 0.73
Standard deviation of the MFD (SD)	5.09 ± 0.13	4.53 ± 0.24	3.84 ± 0.16	4.02 ± 0.25
Coefficient of variation of the MFD (CV, %)	20.39 ± 0.59	20.52 ± 0.79	14.82 ± 0.58	13.06 ± 0.77
Comfort factor (CF, %)	77.79 ± 3.90			
Prickling factor (PF, %)	22.21 ± 3.90			
Fiber share (%)		31.09 ± 2.77	49.80 ± 3.05	19.11 ± 2.80
Medullation (%)	68.91 ± 2.77			

LSM represents the least squares mean, and SE represents the standard error.

The mean fiber diameter of all measured fibers in the sample (MFD), the standard deviation of the mean diameter (SD), the coefficient of variation of the mean diameter (CV), the comfort factor – the percentage of fibers < 30 µm (CF), and the prickling factor – the percentage of fibers > 30 µm (PF) were determined. The mean fiber diameter, the standard deviation of mean diameter, and the coefficient of variation were also determined separately for all of the fiber categories.

The medullation as the percentage share of all medullated fibers, regardless of the medulla type, and the percentage share of the individual fiber categories were determined.

A statistical analysis of the fiber diameter and medullation was performed using SPSS 23.0 software (2016) based on a linear model that included the effect of sex, color variety, and the interaction between sex and color variety. All effects were tested against the residual middle squares to determine the level of significance. P values lower than 0.05 were considered to be statistically significant. The results are presented as the least squares mean (LSM) for each trait along with the standard error (± SE)

Histograms and graphs showing the diameter distribution of all fibers and the category's fiber distribution within the diameter classes were generated.

**Figure 2:**
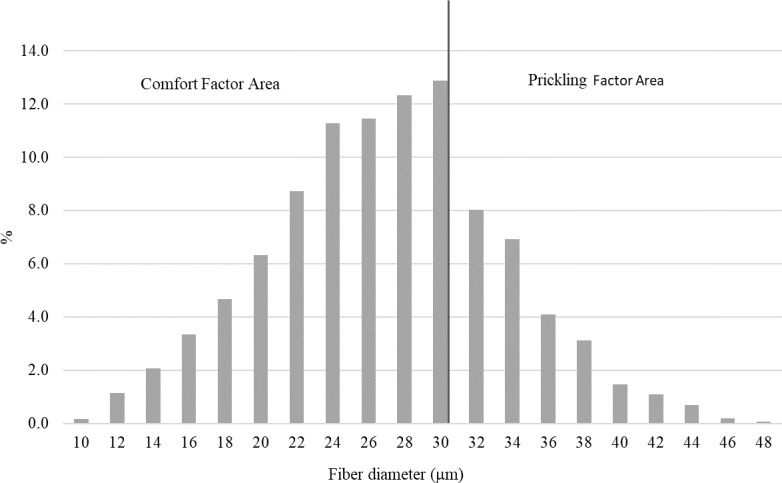
The distribution of the fiber diameter in the wool of the alpacas tested.

## 3 Results and discussion

The mean fiber diameter (MFD) of all alpacas tested was 25.31 µm, and the standard deviation from the mean (SD) and the coefficient of variation (CV) were 5.09 µm and 20.39 %, respectively (Table 1). The thickness variation in all tested samples indicates the fleece uniformity of the alpacas investigated (Fig. 2), which is consistent with their widespread recognition as an animal with uniform wool (Hoffman, 2006; McColl et al., 2004; DeBusk, 2003). The comfort factor (CF) and the prickling factor (PF) were 77.79 % and 22.21 %, respectively.

The wool of the Huacaya alpacas studied in Poland was thicker than that of Huacaya alpacas tested by Cervantes et al. (2010) and Pinares et al. (2018) in Peru. The latter studies also indicated a higher CF in the wool of Peruvian alpacas (87.73 % and 89.8 %, respectively) compared with the value obtained in the present study. In contrast, Valbonesi et al. (2010) determined higher fiber diameter values and coefficients of variation in the wool of Peruvian alpacas (27.41 µm and 36.65%, respectively).

McGregor and Butler (2004) and McGregor (2006) also obtained a higher mean fiber diameter value in most of the Huacaya alpacas used in their studies, which were kept in Australia. Similarly, a higher mean thickness value (from 28.0 to 31.9 µm) was obtained by Wuliji (2000) in alpacas from New Zealand compared with the value reported in this work. In the study of Lupton et al. (2006), the average thickness of wool fibers of alpacas kept in the USA was higher (27.85 µm) than that obtained for the alpacas tested in this study, whereas the CF was lower (68.39 %). It should be noted that the quality of yarn produced by processing alpaca wool is strongly correlated with both the softness and the prickliness, which are both related to the average thickness and the proportion of fibers with a diameter < 30 µm (expressed as the comfort factor). Therefore, it is reasonable to attempt to obtain fibers with a lower thickness; due to the medium to high heritability of this trait, it is possible to achieve this through selective breeding (Gutiérrez et al., 2009; Cervantes et al., 2010; Wuliji, 2000; Ponzoni et al., 1999).

**Figure 3:**
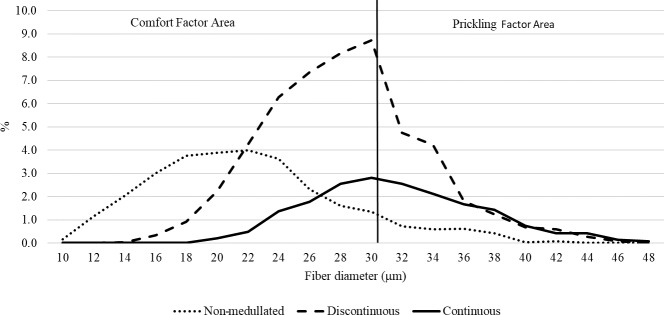
The distribution of each fiber category in the wool of the alpacas tested.

**Figure 4:**
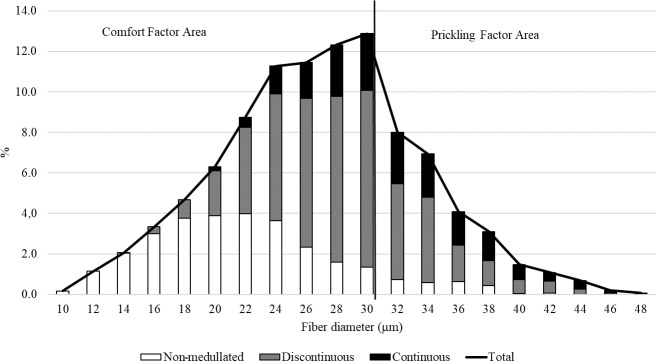
The fiber category distribution within the fiber diameter classes in the wool of the alpacas tested.

The medullation percentage in the wool of the test animals was 68.91 %, the largest share (reaching almost 50 %) of which was recorded for discontinuous medullated fibers. Among all categories of fibers, non-medullated fibers were found to be the thinnest, followed by discontinuous and continuous medullated fibers (Table 1). The differences in the thickness of the types of fibers tested are confirmed by their percentage share in the individual thickness classes (Figs. 3, 4). It is worth noting that the medullas (discontinuous and continuous) were present in both fibers with a diameter > 30 µm and fibers with a diameter < 30 µm (Fig. 4). The presence of medullas in the fibers, in addition to their thickness, may also be responsible for the occurrence of the prickling sensation from knitted and woven fabrics made from alpaca wool (Frank et al., 2014).

Similar total medullation percentage results (67.4 % from their study vs. 68.91 % from this work) were obtained by Pinares et al. (2018) in the study of wool from Peruvian alpacas using the MP (projection microscope) method. The share of non-medullated fibers in Pinares et al. (2018) was also similar to this work, whereas a higher proportion of continuous medullated fibers was recorded (24.1 % from their study vs. 19.11 % from this work). To evaluate the medullation of alpaca wool, the OFDA 100 method is commonly used, but it does not allow for the determination of the different types of medullas. Thus, most available data in the literature refer to the degree of total medullation without considering the medulla category of the fiber.

**Table 2:** The wool thickness and medullation features based on the sex of the animals. The values in bold font denote P<0.05.

Item	Females	Males	F value	P value
	LSM ± SE	LSM ± SE		
Mean fiber diameter (MFD, µm)	23.46 ± 1.03	27.15 ± 1.10	5.984	**0.026**
Standard deviation of the MFD (SD)	4.72 ± 0.18	5.47 ± 0.19	8.201	**0.011**
Coefficient of variation of the MFD (CV, %)	20.60 ± 0.80	20.18 ± 0.86	0.131	0.722
Comfort factor (CF, %)	84.07 ± 5.33	71.51 ± 5.69	2.595	0.127
Prickling factor (PF, %)	15.93 ± 5.33	28.49 ± 5.69	2.595	0.127
MFD of non-medullated fibers (µm)	20.11 ± 0.96	23.27 ± 1.02	5.090	**0.038**
SD of non-medullated fibers	3.35 ± 0.33	5.70 ± 0.35	23.684	**0.000**
CV of non-medullated fibers (%)	16.73 ± 1.08	24.30 ± 1.15	23.099	**0.000**
MFD of discontinuous medullated fibers	24.52 ± 0.96	27.26 ± 1.03	3.747	0.071
SD of discontinuous medullated fibers	3.43 ± 0.22	4.25 ± 0.24	6.282	**0.023**
CV of discontinuous medullated fibers (%)	13.93 ± 0.79	15.71 ± 0.84	2.381	0.142
MFD of continuous medullated fibers (µm)	29.25 ± 0.99	31.81 ± 1.06	3.092	0.098
SD of continuous medullated fibers	3.57 ± 0.35	4.47 ± 0.37	3.165	0.094
CV of continuous medullated fibers (%)	12.15 ± 1.06	13.96 ± 1.13	1.367	0.259
Medullation (%)	60.13 ± 3.79	77.70 ± 4.05	10.032	**0.006**
Continuous medullated fibers (%)	19.30 ± 3.83	18.93 ± 4.10	0.004	0.948
Discontinuous medullated fibers (%)	40.83 ± 4.16	58.77 ± 4.45	8.667	**0.010**
Non-medullated fibers (%)	39.87 ± 3.79	22.30 ± 4.05	10.036	**0.006**

LSM represents the least squares mean, and SE represents the standard error.

The analysis of the fiber thickness parameters based on the sex of the animals indicated that the average fiber diameter was greater in males (P<0.05) than in females. The tested wool of both sexes did not differ in terms of the CV, but the standard deviation of the mean thickness was greater (P<0.05) in the wool of males (Table 2). There were no differences between the wool of the males and females in terms of comfort, which is expressed as the proportion of fibers with a diameter < 30 µm and > 30 µm; although the CF and PF were more favorable in female wool, the differences in the values of these coefficients were not statistically significant (Table 2).

Similar to the present study, Cruz et al. (2017) recorded thicker wool in male Peruvian alpacas, whereas Montes et al. (2008) showed that males alpacas from the Huancavelia region, located between 4100 and 4700 ma.s.l., were characterized by a smaller average fiber diameter compared with females. Wuliji et al. (2000) and Lupton et al. (2006) indicated no differences in wool thickness with respect to sex in their respective studies on alpacas from New Zealand and the USA.

The analysis of fiber thicknesses using different categories shows that the non-medullated fibers in female animals' wool were thinner (P<0.05) compared with fibers of the same category in males. The thicknesses of the non-medullated female fibers were also characterized by better alignment than those from males, as evidenced by lower SD and CV values (P<0.05). There were no differences in the thickness characteristics of the discontinuous medullated fibers, except for the SD value (P<0.05), or the continuous medullated fibers between males and females (Table 2).

**Figure 5:**
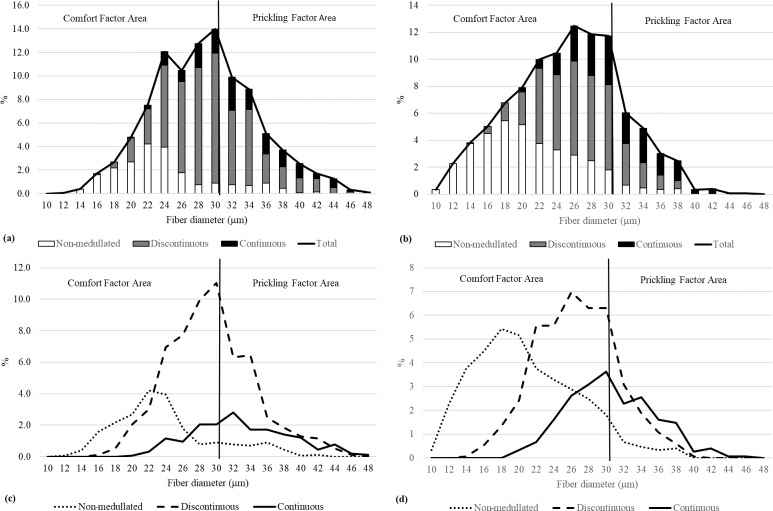
The fiber category distribution within diameter classes in wool from male **(a)** and female **(b)** alpacas, and the distribution of each fiber category in male **(c)** and female **(d)** animals.

The male wool was characterized by a greater degree of medullation (P<0.05) compared with the female wool. In the wool of males, the proportion of discontinuous fibers was greater (P<0.05) and the proportion of non-medullated fibers was lower (P<0.05) than in the wool of females (Table 2). The distribution of the types of fibers examined in the thickness classes indicates a greater proportion of fibers, especially non-medullated fibers, with a diameter < 30 µm in female wool, which may indicate the greater delicacy and softness of wool from female animals. On the other hand, the lower proportion of non-medullated fibers with a diameter < 30 µm in males is compensated for by a greater proportion of fibers with a discontinuous medulla and a smaller proportion of fibers with a continuous medulla compared with females. Although the fiber area > 30 µm is larger in males, it contains a greater proportion of discontinuous fibers and a smaller proportion of continuous fibers compared with females (62.25 % vs. 42.43 %; 30.99 % vs. 50.39 %; Fig. 5a, b). The discontinuous fibers are less stiff, which may reduce the discomfort associated with the prickling factor (Fig. 5).

**Table 3:** The wool thickness and medullation features based on the color of the animals. The values in bold font denote P<0.05.

Item	Light wool	Dark wool	F value	P value
	LSM ± SE	LSM ± SE		
Mean fiber diameter (MFD, µm)	23.45 ± 1.22	27.16 ± 0.89	6.060	**0.026**
Standard deviation of the MFD (SD)	4.83 ± 0.21	5.36 ± 0.15	4.038	0.062
Coefficient of variation of the MFD (CV, %)	21.00 ± 0.95	19.78 ± 0.69	1.068	0.317
Comfort factor (CF, %)	85.13 ± 6.30	70.45 ± 4.59	3.545	0.078
Prickling factor (PF, %)	14.87 ± 6.30	29.55 ± 4.59	3.545	0.078
MFD of non-medullated fibers (µm)	21.19 ± 1.13	22.20 ± 0.83	0.520	0.481
SD of non-medullated fibers	4.80 ± 0.39	4.25 ± 0.28	1.280	0.275
CV of non-medullated fibers (%)	22.00 ± 1.27	19.03 ± 0.93	3.568	0.077
MFD of discontinuous medullated fibers	24.35 ± 1.14	27.43 ± 0.83	4.757	**0.044**
SD of discontinuous medullated fibers	3.63 ± 0.27	4.06 ± 0.19	1.719	0.208
CV of discontinuous medullated fibers (%)	14.87 ± 0.93	14.76 ± 0.68	0.010	0.921
MFD of continuous medullated fibers (µm)	28.79 ± 1.17	32.27 ± 0.86	5.729	**0.029**
SD of continuous medullated fibers	3.42 ± 0.41	4.62 ± 0.30	5.641	**0.030**
CV of continuous medullated fibers (%)	11.76 ± 1.25	14.36 ± 0.91	2.837	0.111
Medullation (%)	58.39 ± 4.48	79.44 ± 3.27	14.391	**0.002**
Continuous medullated fibers (%)	16.90 ± 4.53	21.33 ± 3.30	0.623	0.441
Discontinuous medullated fibers (%)	41.49 ± 4.93	58.11 ± 3.59	7.432	**0.015**
Non-medullated fibers (%)	41.61 ± 4.48	20.56 ± 3.27	14.395	**0.002**

LSM represents the least squares mean, and SE represents the standard error.

The light wool of the study animals was thinner (P<0.05) than the dark wool. The standard deviation and the coefficient of variation remained at a similar level, and the differences between the two color categories in terms of these parameters was not confirmed statistically (Table 3). There were also no statistically significant differences in the CF and PF values, although light wool was characterized by a higher proportion of fibers with a diameter < 30 µm and a lower PF value than dark wool.

Differences in the thickness of the fibers, depending on their category, between the color varieties were noted in relation to the discontinuous and the continuous medullated fibers. The fibers in these categories were thicker (P<0.05) in dark wool than in light wool (Table 3).

Dark wool was also characterized by a higher (P<0.05) degree of medullation. In this wool, the share of discontinuous fibers was higher (P<0.05) and the share of non-medullated fibers was lower (P<0.05) than in light wool. The proportion of continuous medullated fibers did not show statistically significant differences between the color varieties (Table 3).

The results of this study contradict the results obtained by Wuliji (2000) for Huacaya alpacas as well as those obtained by Wurzinger et al. (2006) and Martinez and Rodriguez (1997) for llama wool of various colors; these previous studies indicated that the fiber diameter and the comfort are not dependent on the wool color. Instead, the results of this work are consistent with the conclusions presented by McColl et al. (2004), who indicated that white wool, belonging to the light wool group, is the thinnest. In a similar fashion to the present work, Lupton et al. (2006) and Cruz et al. (2017) found that light fibers had a lower thickness in their respective studies on alpacas kept in the USA and in Huacaya Peruvian alpacas. The present study does not confirm the results of the study by McGregor (2006), which indicates greater medullation in alpacas with white wool.

**Figure 6:**
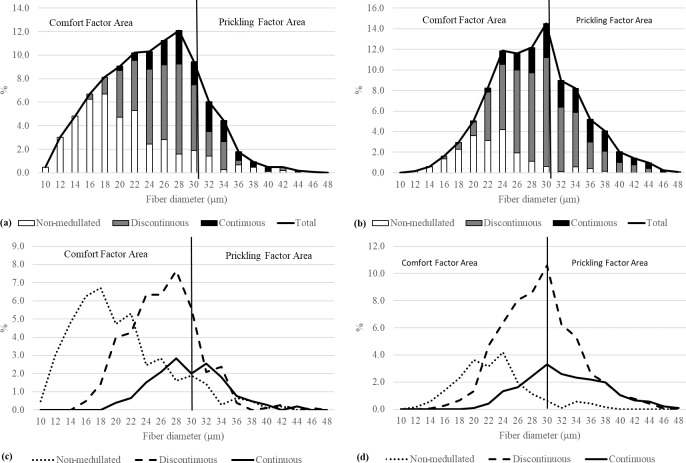
The fiber category distribution within diameter classes in light **(a)** and dark **(b)** wool, and the distribution of each fiber category in light **(c)** and dark **(d)** wool.

A detailed analysis of the fiber category distribution in the thickness classes showed that light wool in the comfort area contained more non-medullated and less discontinuous and continuous fibers compared with dark wool, which may indicate that light fibers are softer (Fig. 6). On the other hand, discontinuous medullated fibers were dominant in the prickling area in dark wool, but the share of fibers with continuous medulla was lower than for light wool (37.36 % vs. 41.83 %). In light wool, more non-medullated fibers with a diameter > 30 µm were recorded than in dark wool (21.57 % vs. 3.92 %; Fig. 6a, b).

The presence or absence of medullas may involve fibers with different thicknesses. Thinner medullated fibers and coarse non-medullated fibers can be produced by secondary hair follicles (Antonini et al., 2004). Regardless of sex and color, discontinuous medulla were observed in 14 µm thick fibers, and non-medullated fibers were found in 48 µm thick fibers. Pinares et al. (2018) noted that the fiber diameter cannot be the only criterion responsible for the prickliness of alpaca wool. Thus, a comfort factor based on fiber diameter may not be a sufficient indicator for discomfort. Hence, when attempting to solve the problem of roughness, stiffness and prickling in alpaca wool clothing, the type of medulla in the fiber should also be considered.

## 4 Conclusions

The wool of the study animals (Huacaya alpacas kept in Poland) showed the character of uniform wool with an average diameter of 25.31 µm, a relatively high proportion of fibers with diameters > 30 µm and medullation of around 69 %. The wool of females was characterized by a lower average fiber diameter and medullation percentage than the wool of males. The wool of both sexes did not differ with respect to the comfort factor or the prickling factor, although the share of non-medullated fibers in the area up to 30 µm was greater in the wool of females than in that of male animals. Non-medullated fibers in female wool were thinner compared with this category of fibers in male wool. Light wool was found to be thinner and to have a smaller share of medullated fibers than dark wool. The discontinuous medullated and continuous medullated fibers in light wools had a smaller average diameter than these categories of fibers in dark wool. Dark wool was characterized by a greater share of discontinuous medullated fibers. The presence of various types of medulla or the absence of medulla was noted in fibers with smaller and larger diameters, regardless of the sex of the animal or color of the wool.

## Data Availability

The data are available from the corresponding author upon request.
